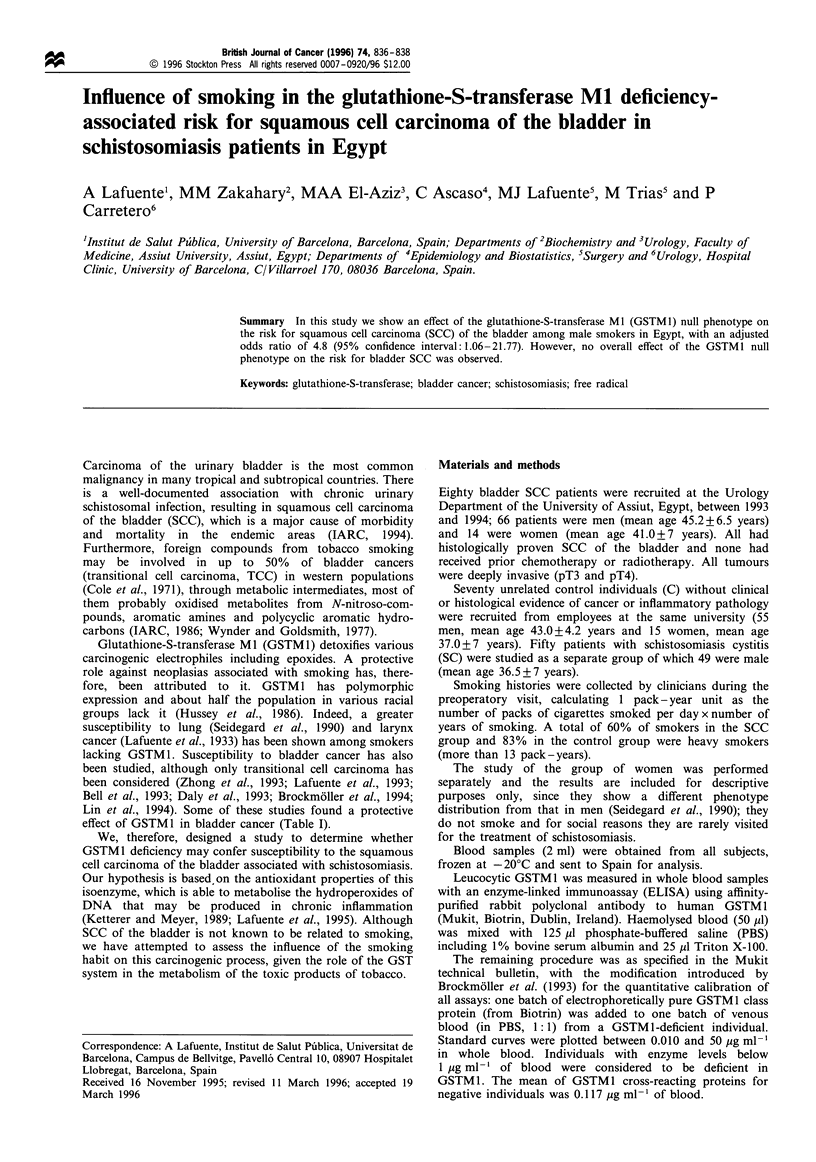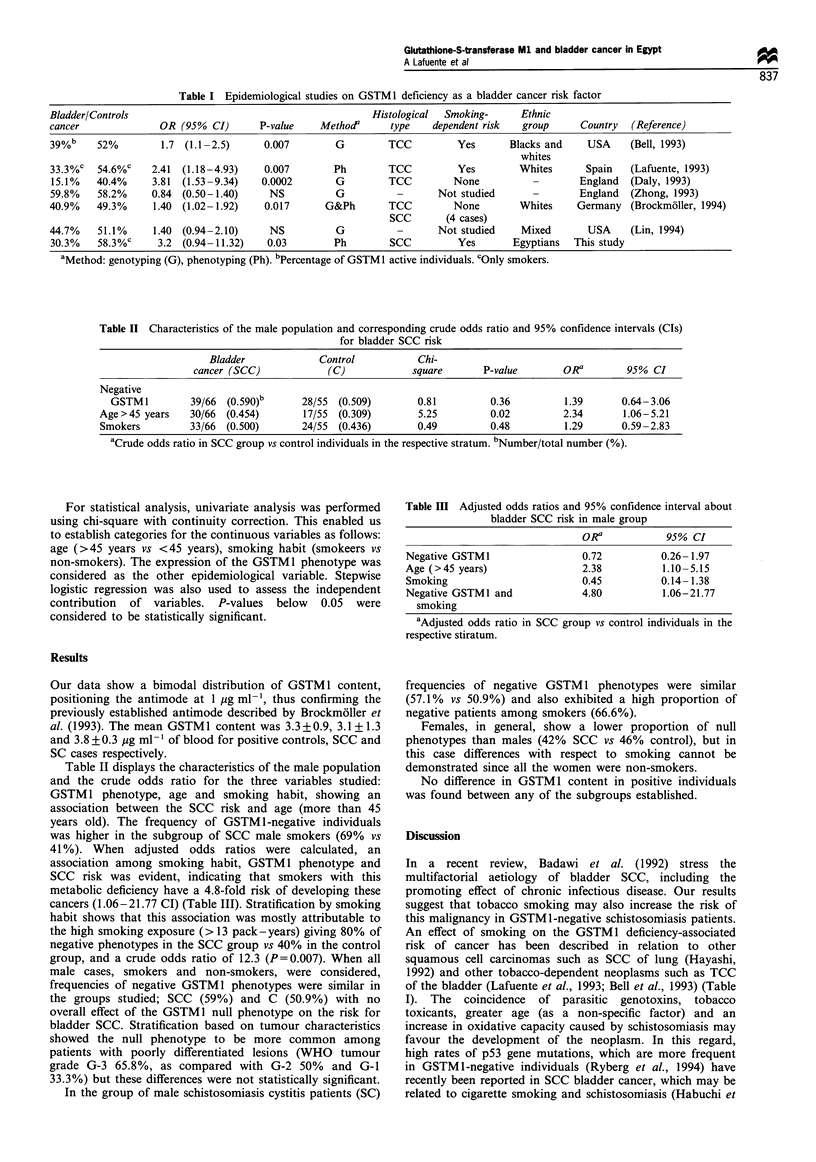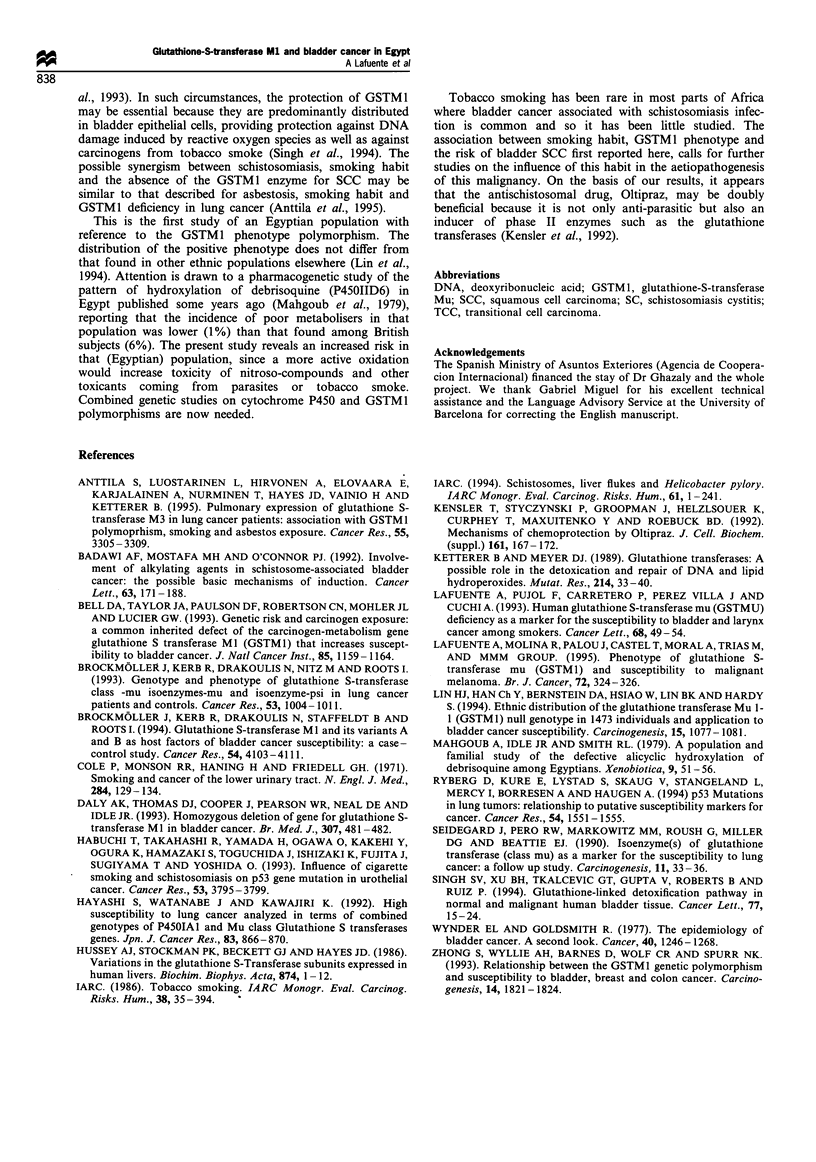# Influence of smoking in the glutathione-S-transferase M1 deficiency--associated risk for squamous cell carcinoma of the bladder in schistosomiasis patients in Egypt.

**DOI:** 10.1038/bjc.1996.445

**Published:** 1996-09

**Authors:** A. Lafuente, M. M. Zakahary, M. A. el-Aziz, C. Ascaso, M. J. Lafuente, M. Trias, P. Carretero

**Affiliations:** Institut de Salut Pública, University of Barcelona, Spain.

## Abstract

In this study we show an effect of the glutathione-S-transferase M1 (GSTM1) null phenotype on the risk for squamous cell carcinoma (SCC) of the bladder among male smokers in Egypt, with an adjusted odds ratio of 4.8 (95% confidence interval: 1.06-21.77). However, no overall effect of the GSTM1 null phenotype on the risk for bladder SCC was observed.


British Journal of Cancer (1996) 74, 836–838
© 1996 Stockton Press All rights reserved 0007-0920/96 $12.00

### Influence of smoking in the glutathione-S-transferase M1 deficiency-associated risk for squamous cell carcinoma of the bladder in schistosomiasis patients in Egypt

A Lafuente[1], MM Zakahary[2], MAA El-Aziz[3], C Ascaso[4], MJ Lafuente[5], M Trias[5] and P Carretero[6]

[1]*Institut de Salut Pública, University of Barcelona, Barcelona, Spain; Departments of* [2]*Biochemistry and* [3]*Urology, Faculty of Medicine, Assiut University, Assiut, Egypt; Departments of* [4]*Epidemiology and Biostatistics,* [5]*Surgery and* [6]*Urology, Hospital Clinic, University of Barcelona, C/Villarroel 170, 08036 Barcelona, Spain.*

**Summary** In this study we show an effect of the glutathione-S-transferase M1 (GSTM1) null phenotype on the risk for squamous cell carcinoma (SCC) of the bladder among male smokers in Egypt, with an adjusted odds ratio of 4.8 (95% confidence interval: 1.06–21.77). However, no overall effect of the GSTM1 null phenotype on the risk for bladder SCC was observed.

**Keywords:** glutathione-S-transferase; bladder cancer; schistosomiasis; free radical

Carcinoma of the urinary bladder is the most common malignancy in many tropical and subtropical countries. There is a well-documented association with chronic urinary schistosomal infection, resulting in squamous cell carcinoma of the bladder (SCC), which is a major cause of morbidity and mortality in the endemic areas (IARC, 1994). Furthermore, foreign compounds from tobacco smoking may be involved in up to 50% of bladder cancers (transitional cell carcinoma, TCC) in western populations (Cole *et al.*, 1971), through metabolic intermediates, most of them probably oxidised metabolites from *N*-nitroso-compounds, aromatic amines and polycyclic aromatic hydrocarbons (IARC, 1986; Wynder and Goldsmith, 1977).

Glutathione-S-transferase M1 (GSTM1) detoxifies various carcinogenic electrophiles including epoxides. A protective role against neoplasias associated with smoking has, therefore, been attributed to it. GSTM1 has polymorphic expression and about half the population in various racial groups lack it (Hussey *et al.*, 1986). Indeed, a greater susceptibility to lung (Seidegard *et al.*, 1990) and larynx cancer (Lafuente *et al.*, 1933) has been shown among smokers lacking GSTM1. Susceptibility to bladder cancer has also been studied, although only transitional cell carcinoma has been considered (Zhong *et al.*, 1993; Lafuente *et al.*, 1993; Bell *et al.*, 1993; Daly *et al.*, 1993; Brockmöller *et al.*, 1994; Lin *et al.*, 1994). Some of these studies found a protective effect of GSTM1 in bladder cancer (Table I).

We, therefore, designed a study to determine whether GSTM1 deficiency may confer susceptibility to the squamous cell carcinoma of the bladder associated with schistosomiasis. Our hypothesis is based on the antioxidant properties of this isoenzyme, which is able to metabolise the hydroperoxides of DNA that may be produced in chronic inflammation (Ketterer and Meyer, 1989; Lafuente *et al.*, 1995). Although SCC of the bladder is not known to be related to smoking, we have attempted to assess the influence of the smoking habit on this carcinogenic process, given the role of the GST system in the metabolism of the toxic products of tobacco.

Correspondence: A Lafuente, Institut de Salut Pública, Universitat de Barcelona, Campus de Bellvitge, Pavelló Central 10, 08907 Hospitalet Llobregat, Barcelona, Spain
Received 16 November 1995; revised 11 March 1996; accepted 19 March 1996

### Materials and methods

Eighty bladder SCC patients were recruited at the Urology Department of the University of Assiut, Egypt, between 1993 and 1994; 66 patients were men (mean age 45.2±6.5 years) and 14 were women (mean age 41.0±7 years). All had histologically proven SCC of the bladder and none had received prior chemotherapy or radiotherapy. All tumours were deeply invasive (pT3 and pT4).

Seventy unrelated control individuals (C) without clinical or histological evidence of cancer or inflammatory pathology were recruited from employees at the same university (55 men, mean age 43.0±4.2 years and 15 women, mean age 37.0±7 years). Fifty patients with schistosomiasis cystitis (SC) were studied as a separate group of which 49 were male (mean age 36.5±7 years).

Smoking histories were collected by clinicians during the preoperatory visit, calculating 1 pack–year unit as the number of packs of cigarettes smoked per day × number of years of smoking. A total of 60% of smokers in the SCC group and 83% in the control group were heavy smokers (more than 13 pack–years).

The study of the group of women was performed separately and the results are included for descriptive purposes only, since they show a different phenotype distribution from that in men (Seidegard *et al.*, 1990); they do not smoke and for social reasons they are rarely visited for the treatment of schistosomiasis.

Blood samples (2 ml) were obtained from all subjects, frozen at −20°C and sent to Spain for analysis.

Leucocytic GSTM1 was measured in whole blood samples with an enzyme-linked immunoassay (ELISA) using affinity-purified rabbit polyclonal antibody to human GSTM1 (Mukit, Biotrin, Dublin, Ireland). Haemolysed blood (50 $\mu$l) was mixed with 125 $\mu$l phosphate-buffered saline (PBS) including 1% bovine serum albumin and 25 $\mu$l Triton X-100.

The remaining procedure was as specified in the Mukit technical bulletin, with the modification introduced by Brockmöller *et al.* (1993) for the quantitative calibration of all assays: one batch of electrophoretically pure GSTM1 class protein (from Biotrin) was added to one batch of venous blood (in PBS, 1:1) from a GSTM1-deficient individual. Standard curves were plotted between 0.010 and 50 $\mu g\ ml^{-1}$ in whole blood. Individuals with enzyme levels below 1 $\mu g\ ml^{-1}$ of blood were considered to be deficient in GSTM1. The mean of GSTM1 cross-reacting proteins for negative individuals was 0.117 $\mu g\ ml^{-1}$ of blood.

Table I Epidemiological studies on GSTM1 deficiency as a bladder cancer risk factor

| *Bladder cancer* | *Controls* | *OR (95% CI)* | P-*value* | *Method*[a] | *Histological type* | *Smoking-dependent risk* | *Ethnic group* | *Country* | *(Reference)* |
|---|---|---|---|---|---|---|---|---|---|
| 39%[b] | 52% | 1.7 (1.1–2.5) | 0.007 | G | TCC | Yes | Blacks and whites | USA | (Bell, 1993) |
| 33.3%[c] | 54.6%[c] | 2.41 (1.18–4.93) | 0.007 | Ph | TCC | Yes | Whites | Spain | (Lafuente, 1993) |
| 15.1% | 40.4% | 3.81 (1.53–9.34) | 0.0002 | G | TCC | None | – | England | (Daly, 1993) |
| 59.8% | 58.2% | 0.84 (0.50–1.40) | NS | G | – | Not studied | – | England | (Zhong, 1993) |
| 40.9% | 49.3% | 1.40 (1.02–1.92) | 0.017 | G&Ph | TCC SCC | None (4 cases) | Whites | Germany | (Brockmöller, 1994) |
| 44.7% | 51.1% | 1.40 (0.94–2.10) | NS | G | – | Not studied | Mixed | USA | (Lin, 1994) |
| 30.3% | 58.3%[c] | 3.2 (0.94–11.32) | 0.03 | Ph | SCC | Yes | Egyptians | This study | |

[a]Method: genotyping (G), phenotyping (Ph). [b]Percentage of GSTM1 active individuals. [c]Only smokers.

Table II Characteristics of the male population and corresponding crude odds ratio and 95% confidence intervals (CIs) for bladder SCC risk

| | *Bladder cancer (SCC)* | *Control (C)* | *Chi-square* | P-*value* | *OR*[a] | *95% CI* |
|---|---|---|---|---|---|---|
| Negative GSTM1 | 39/66 (0.590)[b] | 28/55 (0.509) | 0.81 | 0.36 | 1.39 | 0.64–3.06 |
| Age > 45 years | 30/66 (0.454) | 17/55 (0.309) | 5.25 | 0.02 | 2.34 | 1.06–5.21 |
| Smokers | 33/66 (0.500) | 24/55 (0.436) | 0.49 | 0.48 | 1.29 | 0.59–2.83 |

[a]Crude odds ratio in SCC group *vs* control individuals in the respective stratum. [b]Number/total number (%).

For statistical analysis, univariate analysis was performed using chi-square with continuity correction. This enabled us to establish categories for the continuous variables as follows: age (>45 years *vs* <45 years), smoking habit (smokeers *vs* non-smokers). The expression of the GSTM1 phenotype was considered as the other epidemiological variable. Stepwise logistic regression was also used to assess the independent contribution of variables. *P*-values below 0.05 were considered to be statistically significant.

Table III Adjusted odds ratios and 95% confidence interval about bladder SCC risk in male group

| | *OR*[a] | *95% CI* |
|---|---|---|
| Negative GSTM1 | 0.72 | 0.26–1.97 |
| Age (>45 years) | 2.38 | 1.10–5.15 |
| Smoking | 0.45 | 0.14–1.38 |
| Negative GSTM1 and smoking | 4.80 | 1.06–21.77 |

[a]Adjusted odds ratio in SCC group *vs* control individuals in the respective stiratum.

### Results

Our data show a bimodal distribution of GSTM1 content, positioning the antimode at 1 $\mu$g ml$^{-1}$, thus confirming the previously established antimode described by Brockmöller *et al.* (1993). The mean GSTM1 content was $3.3 \pm 0.9$, $3.1 \pm 1.3$ and $3.8 \pm 0.3$ $\mu$g ml$^{-1}$ of blood for positive controls, SCC and SC cases respectively.

Table II displays the characteristics of the male population and the crude odds ratio for the three variables studied: GSTM1 phenotype, age and smoking habit, showing an association between the SCC risk and age (more than 45 years old). The frequency of GSTM1-negative individuals was higher in the subgroup of SCC male smokers (69% *vs* 41%). When adjusted odds ratios were calculated, an association among smoking habit, GSTM1 phenotype and SCC risk was evident, indicating that smokers with this metabolic deficiency have a 4.8-fold risk of developing these cancers (1.06–21.77 CI) (Table III). Stratification by smoking habit shows that this association was mostly attributable to the high smoking exposure (>13 pack–years) giving 80% of negative phenotypes in the SCC group *vs* 40% in the control group, and a crude odds ratio of 12.3 ($P$=0.007). When all male cases, smokers and non-smokers, were considered, frequencies of negative GSTM1 phenotypes were similar in the groups studied; SCC (59%) and C (50.9%) with no overall effect of the GSTM1 null phenotype on the risk for bladder SCC. Stratification based on tumour characteristics showed the null phenotype to be more common among patients with poorly differentiated lesions (WHO tumour grade G-3 65.8%, as compared with G-2 50% and G-1 33.3%) but these differences were not statistically significant.

In the group of male schistosomiasis cystitis patients (SC) frequencies of negative GSTM1 phenotypes were similar (57.1% *vs* 50.9%) and also exhibited a high proportion of negative patients among smokers (66.6%).

Females, in general, show a lower proportion of null phenotypes than males (42% SCC *vs* 46% control), but in this case differences with respect to smoking cannot be demonstrated since all the women were non-smokers.

No difference in GSTM1 content in positive individuals was found between any of the subgroups established.

### Discussion

In a recent review, Badawi *et al.* (1992) stress the multifactorial aetiology of bladder SCC, including the promoting effect of chronic infectious disease. Our results suggest that tobacco smoking may also increase the risk of this malignancy in GSTM1-negative schistosomiasis patients. An effect of smoking on the GSTM1 deficiency-associated risk of cancer has been described in relation to other squamous cell carcinomas such as SCC of lung (Hayashi, 1992) and other tobacco-dependent neoplasms such as TCC of the bladder (Lafuente *et al.*, 1993; Bell *et al.*, 1993) (Table I). The coincidence of parasitic genotoxins, tobacco toxicants, greater age (as a non-specific factor) and an increase in oxidative capacity caused by schistosomiasis may favour the development of the neoplasm. In this regard, high rates of p53 gene mutations, which are more frequent in GSTM1-negative individuals (Ryberg *et al.*, 1994) have recently been reported in SCC bladder cancer, which may be related to cigarette smoking and schistosomiasis (Habuchi *et*

al., 1993). In such circumstances, the protection of GSTM1 may be essential because they are predominantly distributed in bladder epithelial cells, providing protection against DNA damage induced by reactive oxygen species as well as against carcinogens from tobacco smoke (Singh et al., 1994). The possible synergism between schistosomiasis, smoking habit and the absence of the GSTM1 enzyme for SCC may be similar to that described for asbestosis, smoking habit and GSTM1 deficiency in lung cancer (Anttila et al., 1995).

This is the first study of an Egyptian population with reference to the GSTM1 phenotype polymorphism. The distribution of the positive phenotype does not differ from that found in other ethnic populations elsewhere (Lin et al., 1994). Attention is drawn to a pharmacogenetic study of the pattern of hydroxylation of debrisoquine (P450IID6) in Egypt published some years ago (Mahgoub et al., 1979), reporting that the incidence of poor metabolisers in that population was lower (1%) than that found among British subjects (6%). The present study reveals an increased risk in that (Egyptian) population, since a more active oxidation would increase toxicity of nitroso-compounds and other toxicants coming from parasites or tobacco smoke. Combined genetic studies on cytochrome P450 and GSTM1 polymorphisms are now needed.

Tobacco smoking has been rare in most parts of Africa where bladder cancer associated with schistosomiasis infection is common and so it has been little studied. The association between smoking habit, GSTM1 phenotype and the risk of bladder SCC first reported here, calls for further studies on the influence of this habit in the aetiopathogenesis of this malignancy. On the basis of our results, it appears that the antischistosomal drug, Oltipraz, may be doubly beneficial because it is not only anti-parasitic but also an inducer of phase II enzymes such as the glutathione transferases (Kensler et al., 1992).

**Abbreviations**

DNA, deoxyribonucleic acid; GSTM1, glutathione-S-transferase Mu; SCC, squamous cell carcinoma; SC, schistosomiasis cystitis; TCC, transitional cell carcinoma.

**Acknowledgements**

The Spanish Ministry of Asuntos Exteriores (Agencia de Cooperacion Internacional) financed the stay of Dr Ghazaly and the whole project. We thank Gabriel Miguel for his excellent technical assistance and the Language Advisory Service at the University of Barcelona for correcting the English manuscript.

### References

ANTTILA S, LUOSTARINEN L, HIRVONEN A, ELOVAARA E, KARJALAINEN A, NURMINEN T, HAYES JD, VAINIO H AND KETTERER B. (1995). Pulmonary expression of glutathione S-transferase M3 in lung cancer patients: association with GSTM1 polymoprhism, smoking and asbestos exposure. *Cancer Res.*, **55**, 3305–3309.

BADAWI AF, MOSTAFA MH AND O'CONNOR PJ. (1992). Involvement of alkylating agents in schistosome-associated bladder cancer: the possible basic mechanisms of induction. *Cancer Lett.*, **63**, 171–188.

BELL DA, TAYLOR JA, PAULSON DF, ROBERTSON CN, MOHLER JL AND LUCIER GW. (1993). Genetic risk and carcinogen exposure: a common inherited defect of the carcinogen-metabolism gene glutathione S transferase M1 (GSTM1) that increases susceptibility to bladder cancer. *J. Natl Cancer Inst.*, **85**, 1159–1164.

BROCKMÖLLER J, KERB R, DRAKOULIS N, NITZ M AND ROOTS I. (1993). Genotype and phenotype of glutathione S-transferase class -mu isoenzymes-mu and isoenzyme-psi in lung cancer patients and controls. *Cancer Res.*, **53**, 1004–1011.

BROCKMÖLLER J, KERB R, DRAKOULIS N, STAFFELDT B AND ROOTS I. (1994). Glutathione S-transferase M1 and its variants A and B as host factors of bladder cancer susceptibility: a case–control study. *Cancer Res.*, **54**, 4103–4111.

COLE P, MONSON RR, HANING H AND FRIEDELL GH. (1971). Smoking and cancer of the lower urinary tract. *N. Engl. J. Med.*, **284**, 129–134.

DALY AK, THOMAS DJ, COOPER J, PEARSON WR, NEAL DE AND IDLE JR. (1993). Homozygous deletion of gene for glutathione S-transferase M1 in bladder cancer. *Br. Med. J.*, **307**, 481–482.

HABUCHI T, TAKAHASHI R, YAMADA H, OGAWA O, KAKEHI Y, OGURA K, HAMAZAKI S, TOGUCHIDA J, ISHIZAKI K, FUJITA J, SUGIYAMA T AND YOSHIDA O. (1993). Influence of cigarette smoking and schistosomiasis on p53 gene mutation in urothelial cancer. *Cancer Res.*, **53**, 3795–3799.

HAYASHI S, WATANABE J AND KAWAJIRI K. (1992). High susceptibility to lung cancer analyzed in terms of combined genotypes of P450IA1 and Mu class Glutathione S transferases genes. *Jpn. J. Cancer Res.*, **83**, 866–870.

HUSSEY AJ, STOCKMAN PK, BECKETT GJ AND HAYES JD. (1986). Variations in the glutathione S-Transferase subunits expressed in human livers. *Biochim. Biophys. Acta*, **874**, 1–12.

IARC. (1986). Tobacco smoking. *IARC Monogr. Eval. Carcinog. Risks. Hum.*, **38**, 35–394.

IARC. (1994). Schistosomes, liver flukes and *Helicobacter pylory*. *IARC Monogr. Eval. Carcinog. Risks. Hum.*, **61**, 1–241.

KENSLER T, STYCZYNSKI P, GROOPMAN J, HELZLSOUER K, CURPHEY T, MAXUITENKO Y AND ROEBUCK BD. (1992). Mechanisms of chemoprotection by Oltipraz. *J. Cell. Biochem.* (suppl.) **161**, 167–172.

KETTERER B AND MEYER DJ. (1989). Glutathione transferases: A possible role in the detoxication and repair of DNA and lipid hydroperoxides. *Mutat. Res.*, **214**, 33–40.

LAFUENTE A, PUJOL F, CARRETERO P, PEREZ VILLA J AND CUCHI A. (1993). Human glutathione S-transferase mu (GSTMU) deficiency as a marker for the susceptibility to bladder and larynx cancer among smokers. *Cancer Lett.*, **68**, 49–54.

LAFUENTE A, MOLINA R, PALOU J, CASTEL T, MORAL A, TRIAS M, AND MMM GROUP. (1995). Phenotype of glutathione S-transferase mu (GSTM1) and susceptibility to malignant melanoma. *Br. J. Cancer*, **72**, 324–326.

LIN HJ, HAN Ch Y, BERNSTEIN DA, HSIAO W, LIN BK AND HARDY S. (1994). Ethnic distribution of the glutathione transferase Mu 1-1 (GSTM1) null genotype in 1473 individuals and application to bladder cancer susceptibility. *Carcinogenesis*, **15**, 1077–1081.

MAHGOUB A, IDLE JR AND SMITH RL. (1979). A population and familial study of the defective alicyclic hydroxylation of debrisoquine among Egyptians. *Xenobiotica*, **9**, 51–56.

RYBERG D, KURE E, LYSTAD S, SKAUG V, STANGELAND L, MERCY I, BORRESEN A AND HAUGEN A. (1994) p53 Mutations in lung tumors: relationship to putative susceptibility markers for cancer. *Cancer Res.*, **54**, 1551–1555.

SEIDEGARD J, PERO RW, MARKOWITZ MM, ROUSH G, MILLER DG AND BEATTIE EJ. (1990). Isoenzyme(s) of glutathione transferase (class mu) as a marker for the susceptibility to lung cancer: a follow up study. *Carcinogenesis*, **11**, 33–36.

SINGH SV, XU BH, TKALCEVIC GT, GUPTA V, ROBERTS B AND RUIZ P. (1994). Glutathione-linked detoxification pathway in normal and malignant human bladder tissue. *Cancer Lett.*, **77**, 15–24.

WYNDER EL AND GOLDSMITH R. (1977). The epidemiology of bladder cancer. A second look. *Cancer*, **40**, 1246–1268.

ZHONG S, WYLLIE AH, BARNES D, WOLF CR AND SPURR NK. (1993). Relationship between the GSTM1 genetic polymorphism and susceptibility to bladder, breast and colon cancer. *Carcinogenesis*, **14**, 1821–1824.